# Is Dengue Vector Control Deficient in Effectiveness or Evidence?: Systematic Review and Meta-analysis

**DOI:** 10.1371/journal.pntd.0004551

**Published:** 2016-03-17

**Authors:** Leigh R. Bowman, Sarah Donegan, Philip J. McCall

**Affiliations:** 1 Department of Vector Biology, Liverpool School of Tropical Medicine, Liverpool, United Kingdom; 2 Department of Biostatistics, University of Liverpool, Liverpool, United Kingdom; University of California, Irvine, UNITED STATES

## Abstract

**Background:**

Although a vaccine could be available as early as 2016, vector control remains the primary approach used to prevent dengue, the most common and widespread arbovirus of humans worldwide. We reviewed the evidence for effectiveness of vector control methods in reducing its transmission.

**Methodology/Principal Findings:**

Studies of any design published since 1980 were included if they evaluated method(s) targeting *Aedes aegypti* or *Ae*. *albopictus* for at least 3 months. Primary outcome was dengue incidence. Following Cochrane and PRISMA Group guidelines, database searches yielded 960 reports, and 41 were eligible for inclusion, with 19 providing data for meta-analysis. Study duration ranged from 5 months to 10 years. Studies evaluating multiple tools/approaches (23 records) were more common than single methods, while environmental management was the most common method (19 studies). Only 9/41 reports were randomized controlled trials (RCTs). Two out of 19 studies evaluating dengue incidence were RCTs, and neither reported any statistically significant impact. No RCTs evaluated effectiveness of insecticide space-spraying (fogging) against dengue. Based on meta-analyses, house screening significantly reduced dengue risk, OR 0.22 (95% CI 0.05–0.93, *p* = 0.04), as did combining community-based environmental management and water container covers, OR 0.22 (95% CI 0.15–0.32, *p*<0.0001). Indoor residual spraying (IRS) did not impact significantly on infection risk (OR 0.67; 95% CI 0.22–2.11; *p* = 0.50). Skin repellents_,_ insecticide-treated bed nets or traps had no effect (p>0.5), but insecticide aerosols (OR 2.03; 95% CI 1.44–2.86) and mosquito coils (OR 1.44; 95% CI 1.09–1.91) were associated with higher dengue risk (*p* = 0.01). Although 23/41 studies examined the impact of insecticide-based tools, only 9 evaluated the insecticide susceptibility status of the target vector population during the study.

**Conclusions/Significance:**

This review and meta-analysis demonstrate the remarkable paucity of reliable evidence for the effectiveness of any dengue vector control method. Standardised studies of higher quality to evaluate and compare methods must be prioritised to optimise cost-effective dengue prevention.

## Author Summary

Dengue fever has increased dramatically over the past 50 years and today is the most widespread mosquito-borne arboviral disease, affecting nearly half the world’s population in 128 countries. Until the arrival of a vaccine, control of its *Aedes* vectors has been the only method to prevent dengue infection. With dengue outbreaks occurring at increasing frequency and intensity, we undertook a systematic review and meta-analysis of the literature, to evaluate the evidence for effectiveness of vector control strategies currently available. Forty-one studies (from 5 months to 10 years duration) were included in the review. Most studies investigated combinations of approaches but only 9 studies were randomized controlled trials (RCTs). Remarkably, no RCTs evaluated effectiveness against dengue of insecticide space-spraying (outdoor fogging), the main response to dengue outbreaks used worldwide. Nevertheless, there was limited evidence indicating that house screening and to a lesser extent, community-based environmental management with water container covers could reduce risk of dengue infection. However, skin repellents_,_ bed nets and mosquito traps had no effect while insecticide aerosols and mosquito coils were associated with higher dengue risk. However, the quality of the few studies eligible for inclusion was poor overall, and the evidence base is very weak, compromising the knowledge base for making recommendations on delivery of appropriate and effective control. Given this paucity of reliable evidence, standardised studies of higher quality must now be a priority.

## Introduction

Dengue is a viral infection transmitted between humans by *Aedes* mosquitoes. With an estimated 390 million dengue infections occurring every year, and almost half the world’s population exposed to infection with dengue viruses, it is the most widespread mosquito-borne arboviral disease today, affecting 128 countries worldwide [1–3]. The dramatic increase in dengue over the past 50 years can be attributed to a number of factors, ranging from increased urbanization, in-country and international population movement, erratic water supplies and ineffective or unsustainable vector control [4, 5]. The human and economic cost of frequent dengue outbreaks is high, though current Figs are almost certainly underestimates [6–9]. Dengue is showing signs of emergence in more temperate latitudes [10–13] and is a potential threat to many of the international mass-gatherings that are a feature the modern era, such as the FIFA World Cup and the Olympics, or religious gatherings like the Hajj, although their contribution to global spread has never been proven [14, 15].

Until recent advances in vaccine development [16–17], and the approval and potential availability of the first product in 2016 [18], dengue has been unique among the major vector-borne diseases, in that prevention from infection could only be attempted by reducing or eliminating bites by infected vector mosquitoes [19, 20].

Dengue viruses are transmitted primarily by *Aedes aegypti*, a cosmotropical mosquito that thrives in urban environments. It is highly anthropophilic and breeds in small bodies of fresh water, most commonly in the numerous containers found around the home, ranging from water storage drums and overhead tanks to bottles, buckets and discarded waste items [4]. Between blood feeding and oviposition, adult female mosquitoes rest within or close to human dwellings [19]. A second vector, *Aedes albopictus*, was originally confined to Asia, but in recent decades has expanded its global range and contributed to the spread of the chikungunya virus, as well as dengue [21–24].

Control of dengue vectors can be directed against the immature aquatic stages (larvae and pupae) or the adult mosquitoes, with a number of methods available for each approach. Described in detail elsewhere [19, 25], they can be grouped according to whether they target the vector directly (*i*.*e*. aim to kill mosquitoes using insecticides or natural enemies or prevent them from biting using repellents) or indirectly (*e*.*g*. environmental modification or sanitation improvements that reduce potential larval development sites, or house improvements that prevent mosquito entry). Some approaches require skilled staff and/or dedicated resources (*e*.*g*. specialised spraying equipment, insecticides, transport) in order to be delivered effectively in a vertical approach. For others, affected communities, empowered through education and advocacy, can mobilize and mount effective control operations relatively independently via horizontal or community-based efforts. Hence, space-spraying and larviciding require trained personnel to deliver potentially toxic insecticides using specialized equipment and are dependent on vertical municipality-driven programs. In contrast, reductions in potential larval development sites can be achieved with householders and communities taking responsibility, supported by education and social mobilization [19].

In dengue-affected communities worldwide, immature vector populations are targeted through the reduction or elimination of potential larval development sites, typically by collection of purposeless or discarded containers in ‘clean-up’ or environmental management campaigns; functional or useful sites are either covered (water storage containers), drained (gutters or channels) or treated with an appropriate insecticide (usually referred to as ‘larviciding’) or biological control agent (predatory copepods or fish). Identification of, and targeted action towards, ‘productive’ container types (*i*.*e*. those that are assessed as contributing the greatest burden of pupae, relative to other containers in the area) can potentially enable more cost-effective larval control [26,27].

The typical response to dengue outbreaks is to target the adult mosquito population by space-spraying or fogging with insecticide, delivered outside or inside the home, with the aim of severely reducing the vector population at the time of delivery. This method is not designed to deliver persistent insecticide residues on treated surfaces and if the outbreak continues, it must be repeated at intervals that coincide with the vector life cycle [19].

Previously, Erlanger *et al*. (2008) [28] reviewed data on the effectiveness on vector indices of all vector control methods and concluded that integrated vector control was the most effective, while environmental management had minimal impact. Notably, the evidence for impact of outdoor space spraying was limited, though only 1 of the studies included was less than 30 years old (dated from 2015). Two subsequent reviews [29, 30] focused on peri-domestic space spraying and concluded that there was no evidence to support its use in dengue outbreak control, either as a standalone intervention or in combination with other interventions. Horstick *et al*. (2010) [31] also found no evidence for a demonstrable effect of vector control on entomological indices and identified specific weaknesses in funding, management, staffing and community engagement, all of which conspired to lower operational standards and ultimately restrict any likelihood of success. Recent reviews have examined the evidence for the effectiveness of individual methods, including copepods, fish and temephos [32–34].

Today, dengue outbreaks occur at an increasing frequency and intensity in affected communities worldwide and the need for evidence-based selection of the most appropriate interventions has never been greater. What are the best currently available dengue vector control tools, as measured by their impact on dengue infections, and not simply on vector populations? Are previous dengue control failures the result of low operational and management strategies, or are the available tools simply not effective? What evidence exists to provide a basis for evaluating dengue vector control today? To answer these questions and to provide guidance on the most effective strategies currently available to combat dengue, we report here on a systematic review and meta-analysis of the evidence.

## Methods

### Objectives

To systematically review randomized and non-randomized studies to evaluate the evidence of the effectiveness of vector control interventions in reducing a) *Aedes sp*. vector indices and b) human DENV infection and/or disease. The original search was conducted in April 2012 and updated in December 2013 and on 10^th^ January 2015.

### Eligibility criteria

Table 1 displays the eligibility criteria. Only studies that presented data for a minimum duration of 3 months were included (regardless of the frequency of treatments undertaken within that period), as this was considered the minimum period required to demonstrate a sustained impact on the vector population and/or impact on dengue transmission. In addition, only studies published since 1980 were considered eligible for inclusion, for a number of reasons. The period after 1980 saw the expansion in urban populations worldwide, notably in the less developed countries where the ratio of populations in urban and rural regions began to change dramatically [35,36]. This also was the beginning of the ‘globalization’ era, as characterized by steep increases in trans-national and international movement of humans and merchandise, and the time when all four dengue serotypes were reported in every continent, leading to an increase in the frequency and magnitude of dengue outbreaks [5,37,38]. We are familiar with the achievements prior to 1970, such as the ambitious yellow fever programs when *Aedes aegypti* populations were significantly diminished, and indeed eliminated from many cities and large geographic areas throughout Latin America [1,4,5,39]. On balance, it was concluded that the control tools available before the 1980s (*e*.*g*. the highly persistent insecticide DDT) and the settings in which they were carried out, were not pertinent to the challenge of dengue control in urban environments of the 21st century, based on the significant logistical, sociological and epidemiological changes, and the rise in insecticide resistance in vector populations [40,41] that have occurred in many of those countries during the past 35 years.

**Table 1:** Criteria for inclusion or exclusion of studies.

	Inclusion Criteria	Exclusion Criteria
Study design	Any randomised or non-randomised study design.	Review articles or opinion papers
	Primary research and models using empirical data.	Non-empirical research/ modelled data
Mosquitoes	*Aedes aegypti/ albopictus*	All other mosquito spp.
Interventions	Any study where vector control tools (singly or combined) were used for >3 months	
Outcomes	Any study with empirical data reporting dengue incident data and/or entomological indices monitored longitudinally for the duration of the intervention	Entomological data without longitudinal (interval) data capture
	Dengue cases reported either by the study or obtained from external institutions (e.g. hospital records)	Qualitative dengue reports
Other	Papers published from 1980 onwards	Papers published pre-1980

### Outcomes

The primary outcome was dengue incidence (any reported case data; clinical or lab-confirmed/ serologically positive cases); secondary outcomes were a range of vector indices: Breteau Index (BI), House Index (HI), Container Index (CI), tank positivity, number of mosquito adults, pupae per person index (PPPI), presence of *Aedes* immatures and ovitrap positivity rates.

All methods were pre-specified in the review protocol. PRISMA Group guidelines were followed as standard methodologies [42,43].

### Search strategy

The databases WHOLIS, MEDLINE, EMBASE, LILACS and Science Citation Index were searched using the Medical Subject Heading (MeSH) “dengue” followed by the Boolean operator “and” combined with the following ‘free text’ terms “epidemic” and further combined in succession with: ‘threshold’ ‘sentinel’ ‘early warning’ ‘case management’ ‘vector control’ ‘DDSS’ ‘space spraying’ ‘indoor residual spraying’ ‘fogging’ ‘integrated vector management’ ‘IVM’ ‘source reduction’ ‘container’ ‘larvicide’ ‘repellent’ ‘insecticide’ ‘adulticide’ ‘fumigant’ ‘aerial spraying’ ‘dengue decision support system’. The reference list of each of the included studies was also searched, and ‘‘grey literature” (cited unpublished documents) were sought by communication with authors. No limits were placed on year of publication status or language.

### Study selection

Search results were imported into EndNote (EndNote X5, Build 7473). LRB and PJM independently assessed the title and abstract of each record (or the corresponding full article) retrieved by the search for eligibility; any discrepancies were discussed. The full article was retrieved for each eligible study. The study’s investigators were contacted if eligibility was unclear, additional data were unpublished or the article was inaccessible. Each article was scrutinized to detect multiple publications from the same trial; such publications were included as a single study.

### Data extraction

LRB and PJM independently extracted data according to an agreed checklist and differences were discussed. Trial characteristics and risk of bias information were extracted along with outcome data (S1 Table). For each randomized controlled trial, we extracted the number of individuals randomized and the number of individuals analysed for each treatment group. For dichotomous outcomes, we extracted the number of individuals experiencing the event in each treatment group for each study. For continuous outcomes we extracted means and standard deviations (where presented) or medians, interquartile ranges, and ranges. When such data were not reported, we extracted narrative information and tabulated results. For non-randomized studies, we extracted measures of effect, as well as treatment group data.

### Risk of bias assessment

Using a pre-piloted form, LRB and PJM independently assessed risk of bias and discussed any differences (S2 and S3 Tables). For randomized controlled trials we used the Cochrane risk of bias tool and addressed: random sequence generation; allocation concealment; blinding; incomplete outcome data, selective outcome reporting, and other biases [43]. For each component of each trial, a judgment of high, low, or unclear risk of bias was made and the rationale for the judgment was given (S2 Table and S1 Fig). For non-randomized studies, LRB and PJM used the Quality Assessment Tool for Quantitative Studies [44] (S3 Table). This ensured that each study could be ranked according to inherent study design limitations, which included but were not limited to, bias, confounding and blinding.

### Data analyses

Analyses were performed in Review Manager (RevMan Version 5.2. Copenhagen: The Nordic Cochrane Centre, 2012). We extracted the measure of effect and CI from the study reports. Where possible, we stratified analyses by intervention, outcome, measures of effect and study design. For multi-arm trials, data from numerous intervention groups were pooled. We calculated trial-level results (*i*.*e*. MD, RR or OR and standard error [SE]) and pooled them using random-effects inverse-variance meta-analysis to account for large variability present between studies. Results were visualised in forest plots. Sub-group analyses were used to stratify studies that used different and/ or combination interventions.

Heterogeneity was assessed using the I^2^ test statistic, the chi-squared test (*P*<0.01 indicated possible significance) and by visual inspection of the forest plots to identify overlapping confidence intervals. Studies that could not be visualised in forest plots were presented in tables.

When heterogeneity was detected, possible causes were explored using subgroup analyses and predefined covariates.

Subgroup analyses were planned to explore potential sources of heterogeneity (*i*.*e*. effects of seasonality, mosquito species, duration of intervention, coverage), but analyses were not carried out because of the low number of studies available for analysis. For the same reason, sensitivity analyses that excluded studies with a high risk of bias were pre-planned to assess the robustness of results, but were not carried out. Hence, the planned funnel plots were not constructed to explore possible publication biases.

## Results

### Study eligibility results

**Fig 1:**
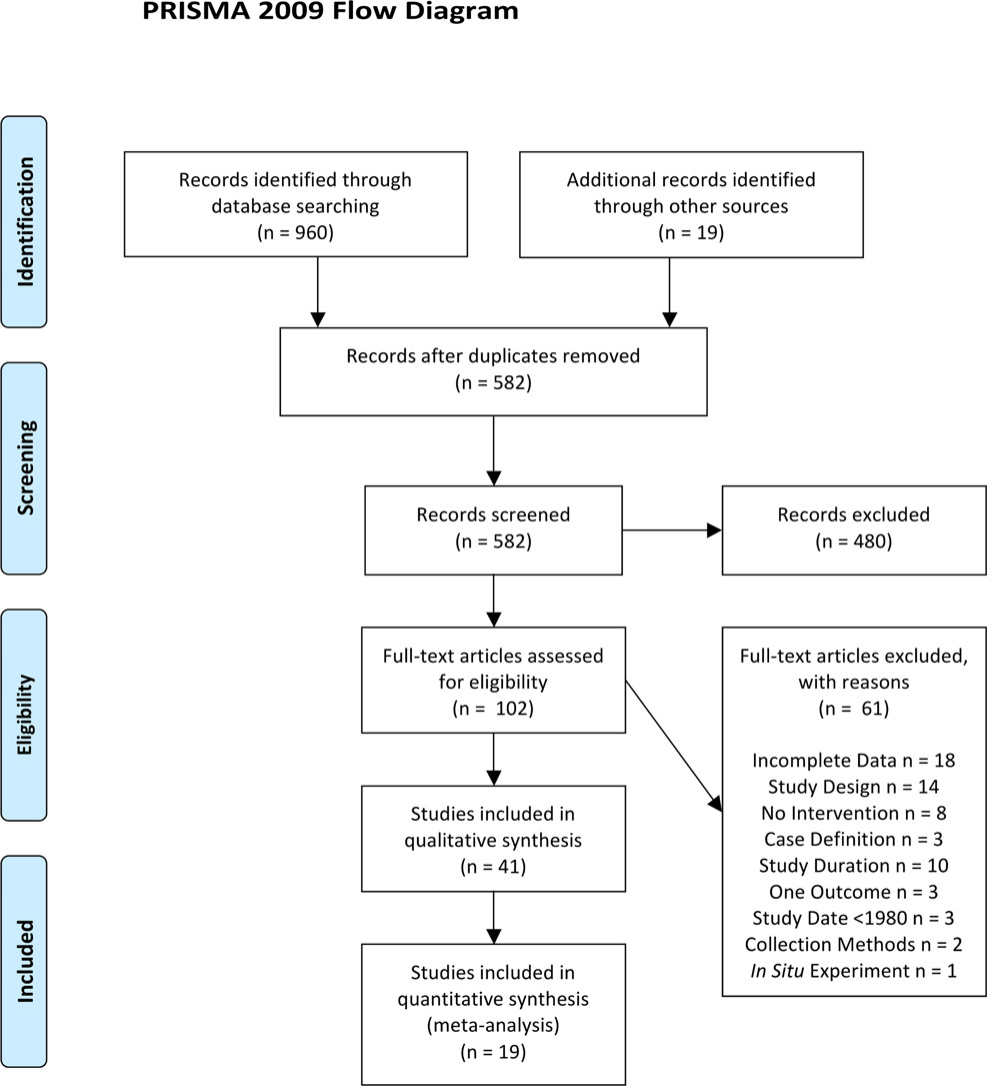
PRISMA 2009 flow diagram. Diagram of searches performed and the number of articles returned and examined at each stage.

A total of 960 potentially relevant studies were identified using systematic searches of the databases, grey literature and their cited reference lists and 19 more were identified from other sources (Fig 1). After removing duplicates, 582 citations were screened, of which 480 were excluded. The full texts of the remaining 102 records were assessed and 61 articles were excluded.

The reasons for exclusion were: incomplete outcome data (18 studies); study was a review, a non-peer reviewed report or a mathematical model (14 studies); no intervention was carried out (eight studies); undefined or inadequate dengue case definition (three studies); intervention or outbreak duration was less than 3 months (10 studies); study included only one required outcome (three studies); study preceded 1980 (three studies); time series data collection not reported (two studies).

Forty-one studies were included in the review [45–85] (S1 Table), nineteen of which reported sufficient data for inclusion in meta-analyses [46–48, 52, 54, 55, 58, 59, 66, 69, 73, 74, 76, 77, 80–83, 85].

### Characteristics of included studies

The main characteristics of included studies are summarised in S4 Table. Of the 41 included studies, geographic study locations comprised: SE Asia (n = 11) or Central America (10), South Asia (8), Australasia (4), South America (5) and North America (3). All studies were published between 1986 and 2014, and 2009 was the median year of publication.

Grouped by study design, the studies comprised: 9 randomised controlled trials (*i*.*e*. 7 cluster-randomized and 2 randomized controlled trials) and 32 non-randomised studies (*i*.*e*. 8 controlled trials, 7 longitudinal studies, 4 interrupted time series studies, 5 before and after studies, 2 observational studies, 1 case-control study, 1 cross sectional study, 1 retrospective observational study, 1 ecological study and 2 models) (S4 Table).

Vertical and community-led interventions were used exclusively in 20 and 10 studies respectively, and 11 studies used a combination of both. Combination interventions (23 studies) were more common than single interventions (18 studies). Study duration ranged from 5 months to 10 years; 16 studies were less than 1 year, 12 took place over 1–3 years and 7 studies were 8 or more years in duration.

**Fig 2:**
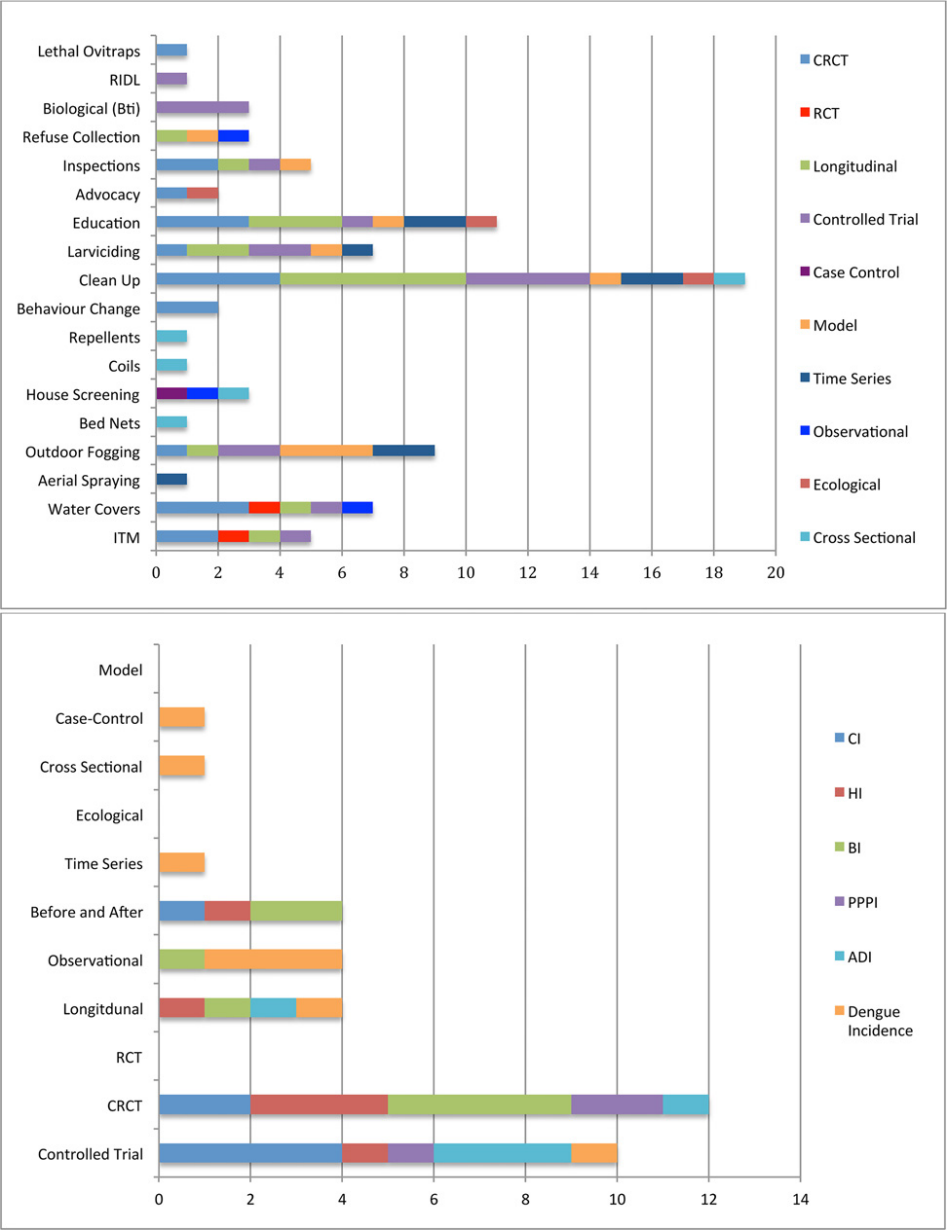
Summary of vector control tools and approaches. Top: Histogram of frequency of interventions reported by the 41 studies, stratified by study design (note that a study design may have evaluated more than 1 intervention). Bottom: Histogram of frequency of reported reductions at p<0.05 stratified by study design (ADI = adult (mosquito) density index; CRCT = cluster randomised controlled trial).

Fig 2 (top) summarises the frequency of vector control tools by study design. The most frequently evaluated interventions were clean-up programs (n = 19), of which 4 were cluster randomised controlled trials. Outdoor fogging (9), education (11), larviciding (7) water jar covers (7) also were the subject of multiple studies.

All studies presented data on *Aedes aegypti*; four presented additional data on *Aedes albopictus* (S4 Table). Nineteen studies reported dengue incidence, 17 studies reported BI, 16 studies reported HI, 11 studies reported CI, 1 study reported tank positivity, 3 studies reported number of mosquito adults, 6 studies reported pupal indices, 3 studies reported ovitrap data.

Fig 2 (bottom) summarises the reported reduction in outcome at a statistically significant level (p<0.05). Of note was the observation that in studies where it was an outcome, dengue incidence was not reduced in either of 2 randomised study designs, although 8 of 14 studies with other experimental designs reported a statistically significant reduction.

### Risk of bias assessment results

#### Randomised studies

The results of this assessment are presented in S2 Table and S1 Fig. Nine studies were at low risk of bias for selective outcome reporting. Seven studies were at low risk of bias for incomplete outcome data, while one was at medium risk and one was at a high risk of bias. There was a high risk of bias due to inadequate blinding in all studies. Risk of bias through allocation concealment was low in one study, unclear in four studies and high in four studies. Risk of bias attributed to generation of allocation sequence was low in four studies, unclear in four studies and high in one study.

#### Non-randomised studies

The results of this assessment for non-randomised studies are shown in S3 Table. Nineteen studies scored 3, equating a weak study, while nine studies scored 2, equal to a moderately strong study, and only two studies scored 1, equal to a strong study.

### Effectiveness of interventions

Nineteen studies [46–48, 52, 54, 55, 58, 59, 66, 69, 73, 74, 76, 77, 80–83, 85] provided sufficient data to allow their inclusion in meta-analyses. The results of those analyses are presented here stratified by reported outcome, either the impact on dengue incidence or on vector indices.

### Impact on dengue incidence

#### Impact of dengue incidence in randomised controlled trials

None of the included reports that investigated the impact of vector control on dengue incidence were randomised controlled studies.

#### Impact on dengue incidence in non-randomised controlled trials

**Fig 3:**
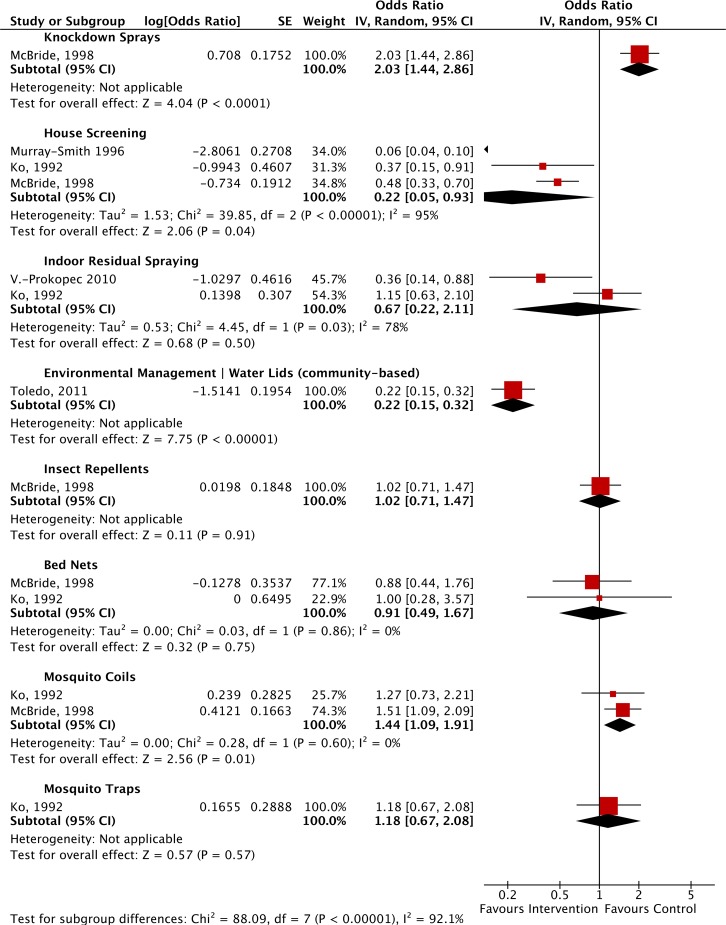
Forest Plot of comparison: Non-randomised controlled trials sub-group analysis stratified by intervention vs. control, for the outcome dengue incidence. *NOTES*: Toledo (2011)[58], original risk ratio was assumed to be similar to the odds ratio, which may bias in favor of the intervention; McBride (1998)[52] cross-sectional study design with no control group); insect repellents upper confidence limit was corrected from 1.44 to 1.47 by RevMan; Ko (1992)[69]: mosquito traps, upper confidence limit was altered by Revman from 2.05 to 2.08; mosquito coils, upper confidence limit altered by RevMan from 2.22 to 2.21; house screens, confidence limit altered by RevMan from 0.89 to 0.91. Vasquez-Prokopec *et al*. (2010)[66], IRS odds ratios relate to secondary dengue infections only.

Five studies measuring the impact of any intervention[s] on dengue incidence using odds ratios were included in one meta-analysis (Fig 3). These included a number of study designs (cross sectional, observational [x2], retrospective observational, case-control) and interventions (knockdown sprays or insecticidal aerosols, house screening, indoor residual spraying, community-based environmental management, insect repellents, bed nets, mosquito coils and mosquito traps). Heterogeneity across the studies was high, most likely due to the varying study designs, number of studies per subgroup and intervention type (I^2^ = 92.1%).

The presence of house screening in homes (three studies: 52,59,69) significantly reduced the odds of dengue incidence compared to homes without screens (0.22: 95% confidence interval (CI) 0.05, 0.93; p = 0.04). Combined community-based environmental management together with the use of water container covers [58] also reduced the odds of dengue incidence to 0.22 (95% CI 0.15, 0.32; p<0.0001).

Indoor residual spraying reduced the odds of infection to 0.67 (95% CI 0.22, 2.11), but the result was not significant (p = 0.50) [66,69]. There was no evidence that the use of mosquito repellents [52]_,_ bed nets [52,69] or mosquito traps [69] significantly increased or reduced the odds of dengue infection, with odds ratios of 1.02 (95% CI 0.71, 1.47; p = 0.91), 0.91 (95% CI 0.49, 1.67; p = 0.75) and 1.18 (95% CI 0.67, 2.08; p = 0.57) respectively.

Conversely, the use of knockdown sprays [52] (OR 2.03; 95% CI 1.44, 2.86) or mosquito coils [52,69] (OR 1.44; 95% CI 1.09, 1.91; p = 0.01) was significantly associated with an increased odds of dengue incidence.

### Impact on vector indices

#### Impact on mosquito indices evaluated in cluster-randomized controlled trials (CRCTs)

**Fig 4:**
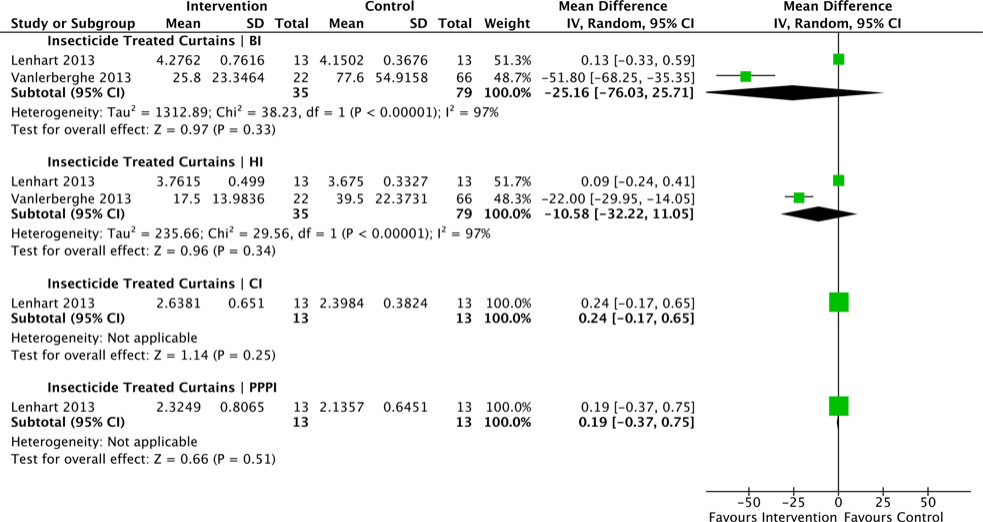
Forest plot of comparison: Cluster randomised controlled trials sub-group analysis for insecticide-treated curtains intervention vs. control for the outcomes Breteau Index, House Index, Container Index and Pupae Per Person Index.

**Fig 5:**
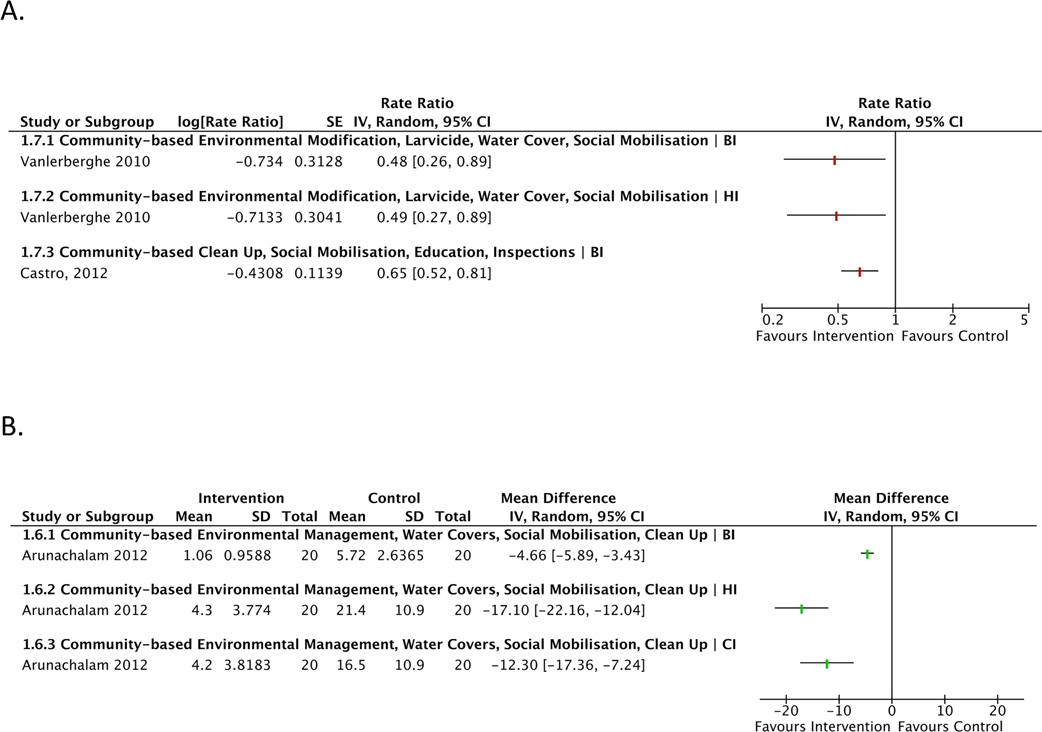
**A. Forest plot of comparison: Cluster randomised controlled trials analysis of community-based environmental management intervention vs. control for the outcomes Breteau Index, House Index**. Cluster Randomised Controlled Trials of community empowerment with routine control vs. control (routine control alone), for the outcome Breteau Index. B. Forest Plot of Comparison: Cluster Randomised Controlled Trials community-based analysis of environmental management intervention vs. control for the outcomes Breteau Index, House Index and Container Index.

Cluster-randomized controlled trials with data suitable for inclusion in these -analyses investigated: insecticide-treated curtains (ITCs) [76, 77]; community-based combination interventions such as waste disposal, clean up campaigns, formation of community working groups, mobilization and education [73]; source reduction, larviciding, entomological surveillance, communication, education and punitive fines [47]. Forest plots of analyses measuring impacts on the BI, HI, CI and pupal indices are shown in Figs 4 and 5.

As shown in Fig 4, ITCs [76, 77] did not significantly reduce the pooled mean difference for either BI, (-25.16; 95% CI -76.02, -25.70; p = 0.33), HI (-10.58; 95% CI -32.22, -11.05; p = 0.34), CI (-0.24; 95% CI -0.16, 0.25) or Pupal indices (-0.19; 95% CI -0.37, 0.75). Heterogeneity between the studies was high, with I_2_ = 97% (p<0.0001) for outcomes BI and HI.

In Cuba, community-based combination interventions significantly impacted BI and HI (Fig 5), with rate ratios of 0.48 (95% CI 0.26, 0.89) and 0.49 (95% CI 0.27, 0.89) in one study [47], while another [83] found that routine interventions led by the community were significantly more effective than routine interventions alone (RR 0.65; 95% CI 0.52, 0.81). Similarly, in an ‘eco-health’ study in India [73], the mean difference was significantly reduced for all metrics: BI -4.66 (-5.89, -3.43), HI -17.10 (-22.16, -12.04) and CI -12.30 (-15.31, -9.29).

#### Impact on mosquito indices evaluated in randomized controlled trials (RCTs)

One study investigated the impact of covering productive larval development container types (S2 Fig). Water tank covers significantly reduced the number of tanks positive for immature stage *Ae*. *aegypti* MD = -4.00 (95% CI -4.96, -3.04) [54], but an impact on dengue incidence was not evaluated.

In quasi-experimental design, larviciding using the insect growth regulator Pyriproxyfen delivered as part of a community-based strategy, was reported to have significantly reduced the rate of dengue incidence in the intervention group: RR 0.19 (95% CI 0.12, 0.30) (S3 Fig) [80].

#### Impact on mosquito indices evaluated in non-RCTs

Although numerous studies evaluated the impact of combinations of interventions on vector populations, it was not possible to combine these into one forest plot, because of the wide range of study designs, outcomes or outcome measures applied. Variously, the studies investigated: clean-up campaigns in conjunction with IRS and larviciding [46]; community-based environmental management (including community-based environmental management, source reduction, larviciding, education, promote formation of CWGs, water covers) [48]; source reduction, larviciding and fogging [55]; nocturnal outdoor fogging [74]; the larval growth inhibitor pyriproxyfen, used alone [80] or in combination with insecticide-treated water covers [82]; lethal ovitraps [81]; release of genetically modified mosquitoes (Release of Insects with Dominant Lethality, RIDL) [85].

Community-based environmental management significantly reduced the House Index: MD = -2.14 (95% CI -3.72, -0.56) [48] (S4 Fig) and combination interventions (clean-up campaigns in conjunction with IRS and larviciding) reduced ovitrap positivity MD = -10.30 (95% CI -12.80, -7.80) [46] (S4 Fig). The use of fogging, source reduction and larviciding resulted in lower odds of detecting increased larval densities (S5 Fig): Breteau Index OR = 0.15 (95% CI 0.10, 0.24) and House Index OR = 0.13 (95% CI 0.08, 0.22) when compared to baseline [55], while the odds of the presence of immature stage *Aedes* were reduced in the intervention group, through the combined use of Olyset net covers for waters jars and pyriproxyfen for a period of 5 months (S2 Fig) [82]. Biogents Sentinel lethal ovitraps demonstrated potential in reducing the number of circulating adult mosquitoes, although this result was modest and not significant: MD 0.30 (95% CI -0.74, 0.13) (S6 Fig) [81].

Outdoor nocturnal ultra-low volume fogging significantly reduced numbers of adult *Ae*. *albopictus* in the intervention group by -13.90 (95% CI -21.86, -5.94) (S4 Fig) but did not measure effects on immature stages [74]. Sampling was conducted using BioGents Sentinel Trap and fogging was conducted between 3–5 times per annum; 43–90% mosquito control was achieved.

In a seminal field study with genetically-modified mosquitoes in the Cayman islands, scheduled releases of sterile male mosquitoes reduced the odds of ovitrap positivity in intervention clusters compared with control clusters: OR 0.11 (95% CI 0.07, 0.18) by [85] (S7 Fig).

## Discussion

The dramatic growth in dengue over the past 35 years has been a remarkable epidemiological event and, as evidenced by its continued global spread, a challenge for which the public health community was not prepared. It is not surprising that 24 of the 41 studies included in this review were published in the past 7 years, reflecting the increase in attention and resources devoted to devising effective control strategies as recognition of the dengue pandemic grew. However, the fact that the global increase in focus on dengue control generated so few studies performed at a standard required for inclusion in this review, indicates that the magnitude of the response to the dengue pandemic has not been sufficient. Moreover, most of these studies investigated the impact of interventions on dengue vector indices alone, rather than dengue incidence. This also is discouraging, as the limitations of the Stegomyia larval indices, primarily their poor correlation with dengue transmission, are well known [86]. Finally, the inadequacy of the response to global dengue threat is demonstrated by the identification of thirteen studies that measured the impact of vector control on dengue incidence in the past 35 years, and that only six of these were suitable for inclusion in a meta-analysis. Simply stated, we do not have a clear understanding of which of the currently available interventions actually work, where or when they succeed or might work best, and the reasons why they succeed or fail.

Nowhere is the inadequacy more apparent than in the absence of appropriately designed trials to evaluate insecticide fogging or space-spraying for the prevention of dengue transmission. Although space spraying is the standard public health response to a dengue outbreak worldwide, and is recommended by WHO for this purpose [19], our study revealed the scant evidence available from studies to evaluate this method sufficiently. Earlier reviews also noted this serious omission from the literature published before 1980 [29,31]. Remarkably, no randomised controlled trials have been undertaken to evaluate the effectiveness of space-spraying or fogging to reduce dengue transmission or dengue incidence, anywhere in the past 35 years. We identified only one study [74] suitable for inclusion in a meta-analysis that demonstrated a significant impact of outdoor fogging on dengue vector populations.

Without adequate evidence, it is impossible to determine how effective space-spraying programs, whether indoor or outdoor, have been. It may be the case that outdoor fogging has the potential to impact on dengue vector populations sufficiently to impact transmission, but the minimum treatment frequency and geographic area requiring treatment remain unknown. The most encouraging report comes from a recent longitudinal study analysing twelve years of data from the city of Iquitos in Peru [84], which concluded that dengue cases could be reduced if intensive city-wide space-spraying (outdoor fogging) was conducted early in the transmission season. Given the cost implications of delivering a similar scale treatment in an even larger city, possibly with the need to do so in advance of an outbreak crisis, further studies to demonstrate the potential benefits are essential.

Of those that could be assessed adequately, the method with the most evidence supporting effectiveness in preventing dengue transmission was house screening. Data from cross-sectional [52] and case-control studies [59] in Australia, and a case-control study in Taiwan (69) were included in a meta-analysis that indicated a significant protective effect of window and door screens on dengue transmission as detected by serology (ELISA or HIA (haemagglutination inhibition assay)) (Fig 3). Although the weaker study designs limited the power of this result, the results are encouraging. *Aedes aegypti* exhibit predominantly indoor resting and blood feeding behaviour (termed endophagic and endophilic behaviour, respectively)[87], and barriers to access would be expected to impact on this species. Malaria vector mosquitoes and other arthropods of medical importance are also active indoors and can be targeted in the same way, increasing the likelihood of perception of benefit and adoption by householders. “Mosquito-proofing” houses was first considered over a century ago, and its potential as a sustainable and effective tool for malaria control has been evaluated in randomized controlled trials in recent years [88–90]. New investigations of screening for dengue prevention are also underway. Recent studies in a high-risk dengue setting in Mexico reported that window and door screens were a popular and widely-adopted intervention that significantly reduced domestic infestations of *Aedes aegypti* [91, 92]. House screening is not included in the current WHO dengue guidelines, but given its potential and wide ranging benefits, it is a strong candidate for randomised controlled trials to evaluate its effectiveness in preventing dengue.

Two observational studies reported on the impact of indoor residual spraying IRS, with contradictory results and while one of these reported a positive significant reduction in the odds of (secondary) incidence [66], the second study reported an insignificant increase [69]. Consequently, the pooled odds ratio showed no statistically significant effect between intervention and control groups. While indoor residual spraying can target *Aedes aegypti*, such methods have rarely been used, nor are currently recommended [19, 93, 94]. Yet IRS is already used widely to control a number of other vector-borne diseases in various settings worldwide and, as it allows the delivery of a range of different insecticide classes, it can be an important tool for managing insecticide resistance [95–98]. The possibility that existing IRS programs might be expanded with minimal change to include dengue is an attractive prospect.

Probably the most widespread practices to suppress dengue vector populations are clean-up campaigns, typically community-driven and in tandem with education and health promotional campaigns as well as numerous additional approaches. Efforts promoting environmental and peri-domestic clean-up to reduce vector larval development sites have been routine practices in many dengue-endemic localities for decades and as shown in Fig 2, they were the most common intervention evaluated in the reviewed studies. However, clean-up campaigns were evaluated only as one element within multiple interventions or they continued to be promoted as a background across all the arms within a study. Thus, source reduction or clean-up campaigns were applied in some way in 20 studies but were associated with interventions ranging from fogging or water container covers targeting adult mosquitoes to larviciding and copepods for control of immatures (S3 Table). Hence it is not possible to dissect their specific contribution to reducing vector populations or their impact on dengue transmission. Of these, the strongest evidence (Fig 3) was from Cuba [58] where results indicated that community working groups (CWGs), initially set up some years earlier, in a preceding study [71] promoting environmental management, conversion of garbage zones into gardens, water pipe repairs and the use of water container covers not only reduced vector indices, but also impacted dengue transmission, significantly more than the routine *A*. *aegypti* control programme. Although WHO recommends community participation as an essential element of sustainable dengue prevention [99], there is little evidence that it can impact on dengue transmission [100]. A number of randomised controlled studies have demonstrated significant impacts on vector indices [47, 48, 73, 83, 101](Fig 5) even though the methods of intervention varied considerably between the studies. Results from a cluster randomised controlled trial in Nicaragua and Mexico [102] reported reductions in dengue sero-conversion rates and self-reported dengue cases as well as vector indices, following community mobilisation to deliver pesticide-free vector control. Clearly further evidence is needed. It remains to be determined how best practice is defined in any setting (*i*.*e*. which tools or methods the community should employ), and what coverage is necessary in order to not simply reduce mosquito indices, but to impact on dengue virus transmission.

The use of fish and crustaceans as biological control agents that prey on or compete with the immature vector stages may have potential in certain contexts, but we identified only three studies that evaluated copepods (aquatic Crustaceans) [78, 79, 103]. In all cases, the crustaceans were used together with clean-up programs, obscuring the impact of each method, and none of the reports provided sufficient data to be included in a meta-analyses. Consistent with earlier specific reviews [32, 34], there remains little evidence to suggest that biological control has widespread potential.

A substantial number of reports demonstrated impacts on vector indices of insecticide-treated materials (ITMs), deployed as window or door curtains [54, 75, 77, 82, 104, 105], although they were effective only where houses with fewer and smaller windows and doors [75–77, 104] and where coverage of the intervention was particularly high [77]. Hence, in the meta-analyses, no significant impact on vector populations was indicated and the heterogeneity between the studies was high (Fig 4). Effects on dengue incidence of ITMs used as vertical window or door screens or as horizontal covers for water containers, need to be quantified in locations and contexts where housing conditions indicate suitability. ITMs, used as curtains hung or fixed tightly across external windows and doors, function in a similar way to mesh screens, and potentially could provide enough protection without the need for insecticide, as suggested by a study in Mexico, where ITMs reduced vector populations even though the targeted population was highly resistant to the insecticide used [90].

There was no evidence of any impact on dengue infection risk by insecticide-treated bed nets [52,69], mosquito traps [69, 81] or mosquito repellents [52]. Ongoing studies are investigating a range of novel trap designs for *Aedes spp*. surveillance and control [106–108] but to date, evidence of traps preventing any mosquito-borne disease remains elusive. Both opinion and evidence are weighed against the use of skin repellents for prevention of vector-borne diseases [109], and attention has moved towards a new generation of spatial repellents, to be deployed within or close to houses to prevent mosquito entry, possibly in combination with attractant lethal traps in what is termed a ‘push-pull’ strategy [108, 110].

The significant negative associations found between the use of insecticide aerosols [52] and mosquito coils [52,69] and higher odds of dengue incidence have a number of possible explanations. These tools may have been purchased in response to an actual increase in mosquito numbers, or a dengue case in the home or a neighbour’s house, during a period of dengue transmission. Alternately, householders using aerosols or coils may have relied solely on these anti-mosquito devices and not have adopted any other more effective preventative measures.

Approaches involving the use of genetically modified (GM) mosquitoes or the intracellular symbiont *Wolbachia* [111] are recent advances in insect control and only one field trial, demonstrating impact on the vector population only [85], was included in this review. An increase in the numbers of reports from ongoing new trials can be expected, although the use of GM mosquitoes for dengue control will have to confront or overcome additional regulatory or ethical challenges and requirements prior to field tests and eventual deployment [112–116].

Regarding trials of methods that require the use of insecticides, we noted that while 23/41 studies examined the impact of insecticide-based tools, only 9 of these cited recent information on insecticide resistance or referred to an evaluation of the susceptibility status of the target vector population at any stage of the study. Resistance to DDT, pyrethroids and other insecticides has been documented widely in dengue vectors, and continues to emerge, potentially impacting on intervention effectiveness [40, 117–119]. Clearly, insecticide susceptibility testing must be an integral part of any trial where insecticide-based interventions are under evaluation, as recommended by the World Health Organisation [4].

Today, there is a widespread perception that *Aedes aegypti* control ‘has failed’ or that existing methods will not reduce dengue transmission, and that this is why we should abandon existing approaches and invest in or pursue alternative strategies [111, 120, 121]. As we have shown in this review and meta-analysis, this is incorrect. In reality, there is very little reliable evidence from appropriately designed trials to reach a conclusion about any of the control methods available. That this also applies to insecticide space-spraying or fogging illustrates clearly the urgent need for such fundamental trials.

Care in designing studies is critical. Randomized controlled trials are the most robust design for evaluating the effectiveness of any intervention [122]. In our review, only eight of the nineteen reports included in the meta-analysis (7 CRCTs, 1 RCT) were randomised, none of which reported a significant impact on dengue incidence. In contrast, eight other studies that reported a positive reduction in dengue incidence at p<0.05, were not derived from randomised controlled trials, but from weaker experimental designs (see Fig 3). Weakness in the designs of trials investigating vector control tools have been recognised, and expert guidance, identification of challenges and pitfalls and clear recommendations for improvement are available [123,124].

Also apparent from this review is the large number of studies investigating impacts on the vector population alone, with no measures of the effectiveness of the intervention on dengue transmission. We recognise that detecting dengue viruses or confirming current, recent or historic dengue infections are not simple routine or inexpensive tasks, requiring skills and equipment that are not available without considerable investment. However, without this additional investment, the value of many studies that are limited to evaluating impacts on the vector alone is seriously reduced. Demonstration of impact on vector populations is achievable and often reported but is no guarantee that an intervention will translate into a reduction in dengue transmission [125, 126]. This is particularly true for dengue, where the complex relationship between vector abundance, virus transmission and human infection rates are far from clear [86,127,128].

As well as their role in dengue transmission, *Aedes aegypti* is the main urban vector of yellow fever in Africa and South America, and this species and *Aedes albopictus* variously are vectors of the Chikungunya and Zika viruses, two emerging human pathogens that constitute a new global threat [129–132]. Despite the fears surrounding these threats, the urge to respond must be tempered by reality, and based on sound evidence. In the large urban zones where these vectors proliferate, to simply continue to use what has always been used, for that reason alone, or to pursue new approaches without sound supporting evidence would be wrong, and potentially a profligate waste of resources. Hence, there is an argument for instituting a global independent advisory body to guide decisions regarding the selection of approaches and tools for control or prevention of infections transmitted by urban *Aedes sp*. vector populations, and the design of appropriate multi-centre trials to evaluate their effectiveness. With this in mind, we hope that the findings of this review and meta-analysis will contribute to the sound evidence base on which that approach would be founded.

## Supporting Information

S1 Checklist PRISMA checklist. (PDF)

S1 Table Data extraction summary for reviewed studies. (XLSX)

S2 Table Cochrane table of bias for randomized controlled trials. (XLSX)

S3 Table Assessment of the validity of reviewed studies: Table of bias and QATQS (quality assessment tool for quantitative studies). (XLSX)

S4 Table Data extraction table for all reviewed studies. (XLSX)

S1 Fig 100% stacked graph of Cochrane Table of bias results. (TIFF)

S2 Fig Forest plot of comparison: Randomised controlled trials of net covers on water storage tanks vs. control for the outcome tank positivity. (TIF)

S3 Fig Forest plot of comparison: Quasi-experimental study on community participation using pyriproxyfen vs. control for the outcome dengue incidence. (TIF)

S4 Fig Forest plot of comparison: Non-randomised controlled trials subgroup analysis for multiple interventions vs. control for the outcomes BGS Adult Catch, Breteau Index and ovitrap positivity. (TIF)

S5 Fig Forest plot of comparison: Non-randomised controlled trials of multiple interventions vs. baseline for the outcomes Breteau Index, House Index. Controlled trial subgroup analysis for larvicide, ULV/ source reduction and Olyset container covers and pyriproxifen vs. control, for the outcomes HI, BI and presence of *Aedes sp*. immatures stages.
(TIF)

S6 Fig Forest plot of comparison: Cluster randomised controlled trials sub-group analysis for BioGents Sentinel Trap vs. control for the outcome number of mosquito adults. (TIF)

S7 Fig Forest plot of comparison: Non-randomised controlled trial on RIDL (release of insects with dominant lethality) *Aedes aegypti* vs. control for the outcome ovitrap positivity. (TIF)
