# Supplementary figures and images for: Is Dengue Vector Control Deficient in Effectiveness or Evidence?: Systematic Review and Meta-analysis
Source: PLoS Negl Trop Dis. 2016 Mar 17;10(3):e0004551. doi: 10.1371/journal.pntd.0004551 (PMC4795802; doi:10.1371/journal.pntd.0004551)

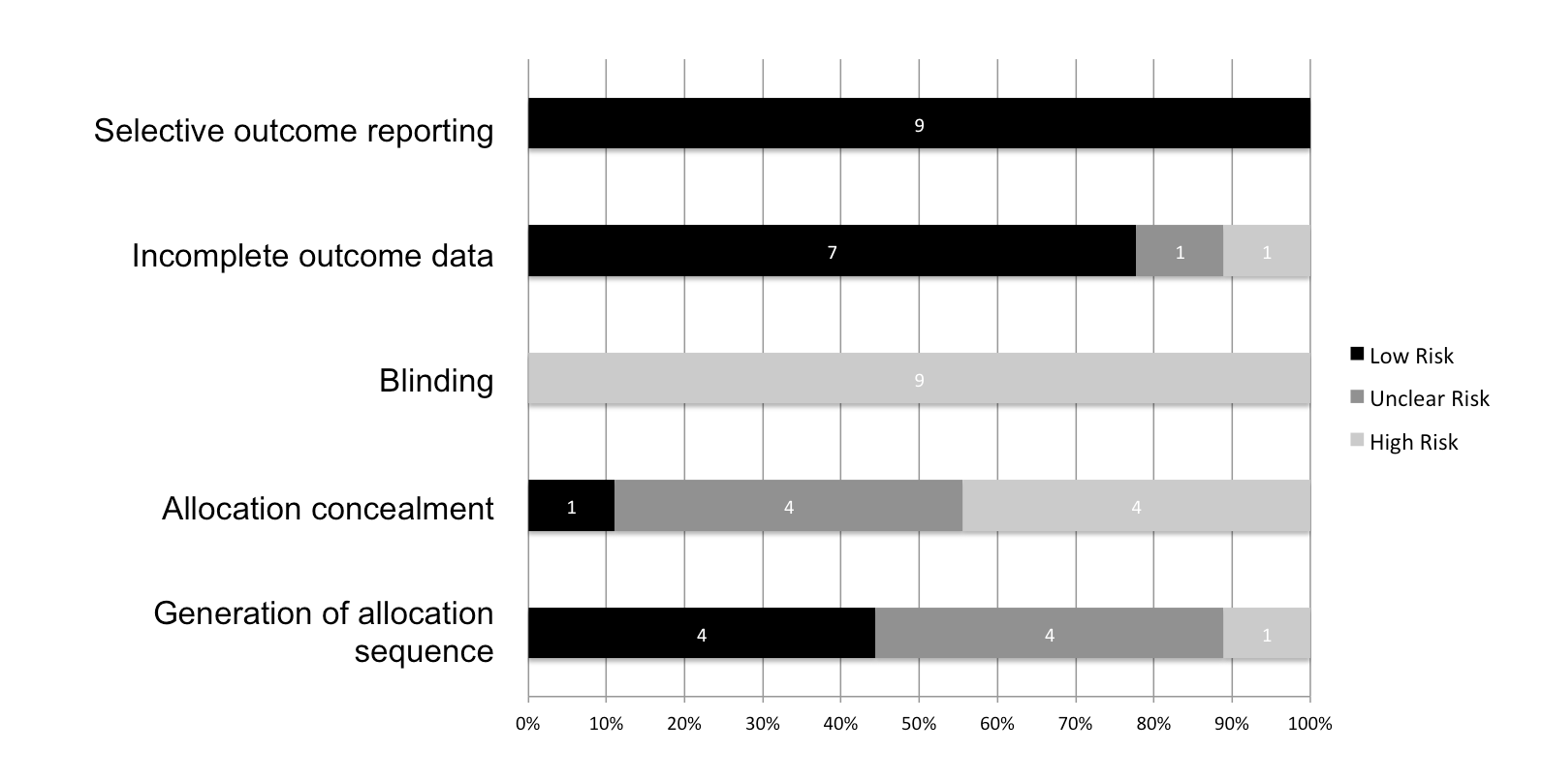

Supplement: S1 Fig — (TIFF) [file pntd.0004551.s006.tiff]

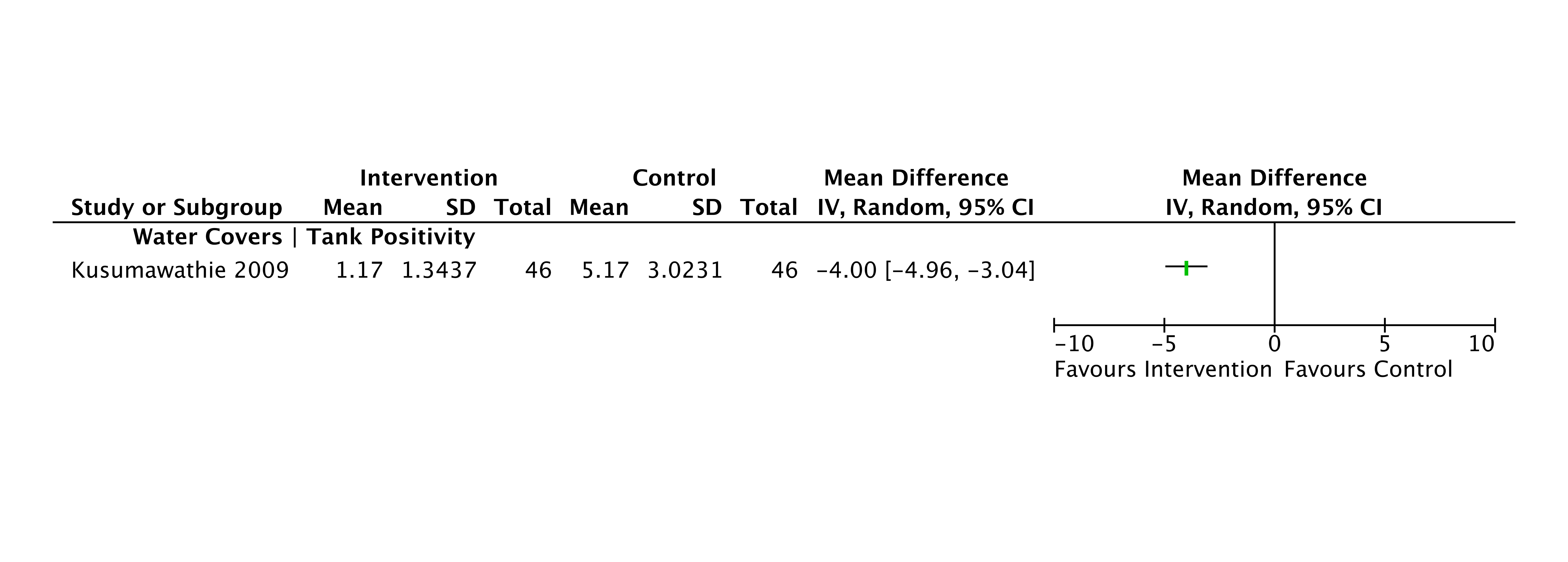

Supplement: S2 Fig — (TIF) [file pntd.0004551.s007.tif]

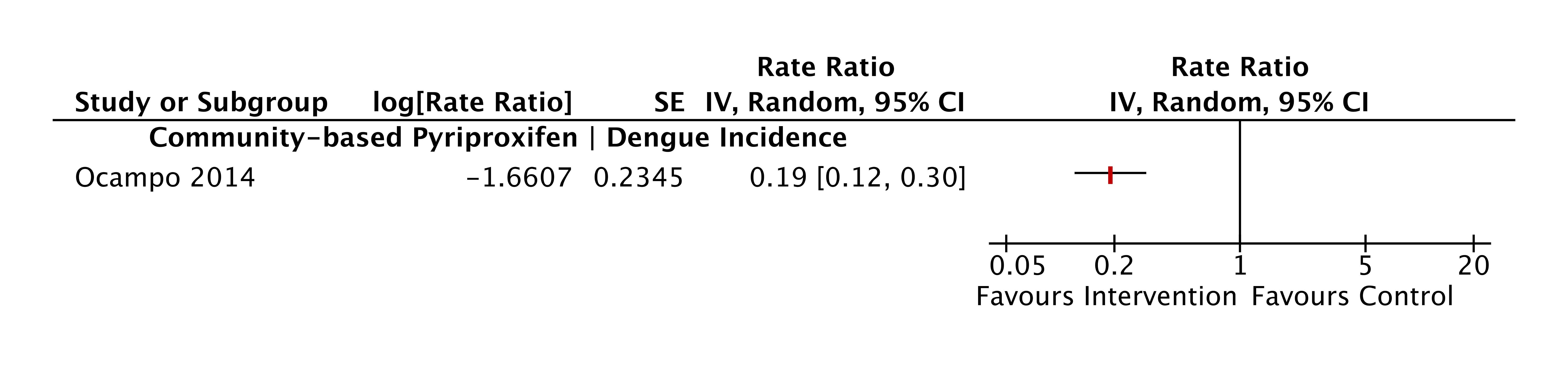

Supplement: S3 Fig — (TIF) [file pntd.0004551.s008.tif]

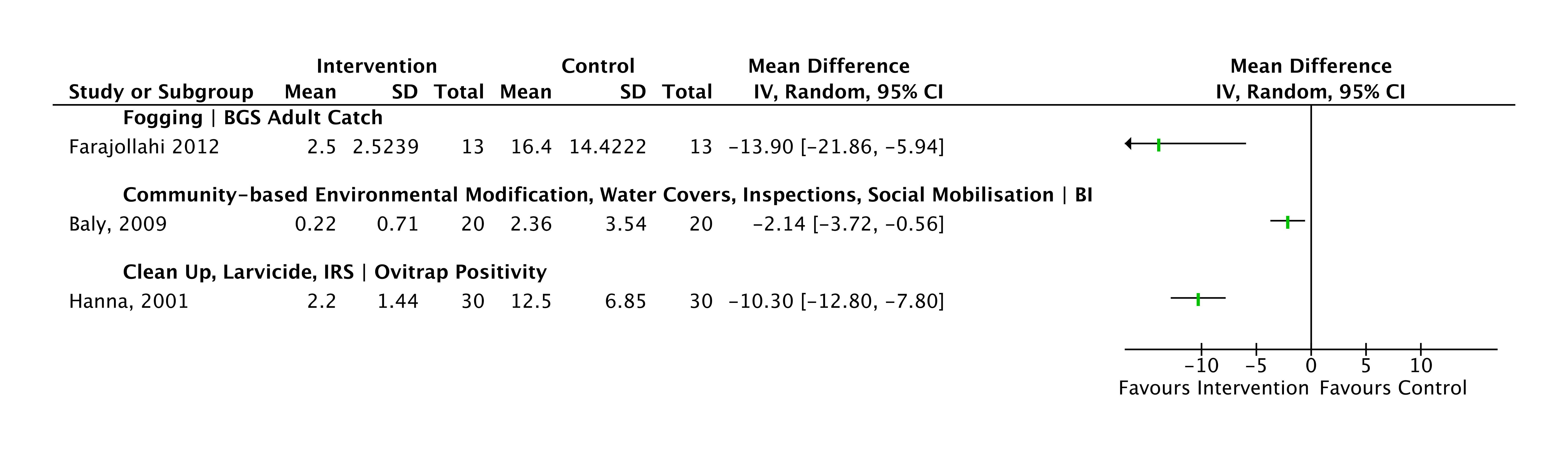

Supplement: S4 Fig — (TIF) [file pntd.0004551.s009.tif]

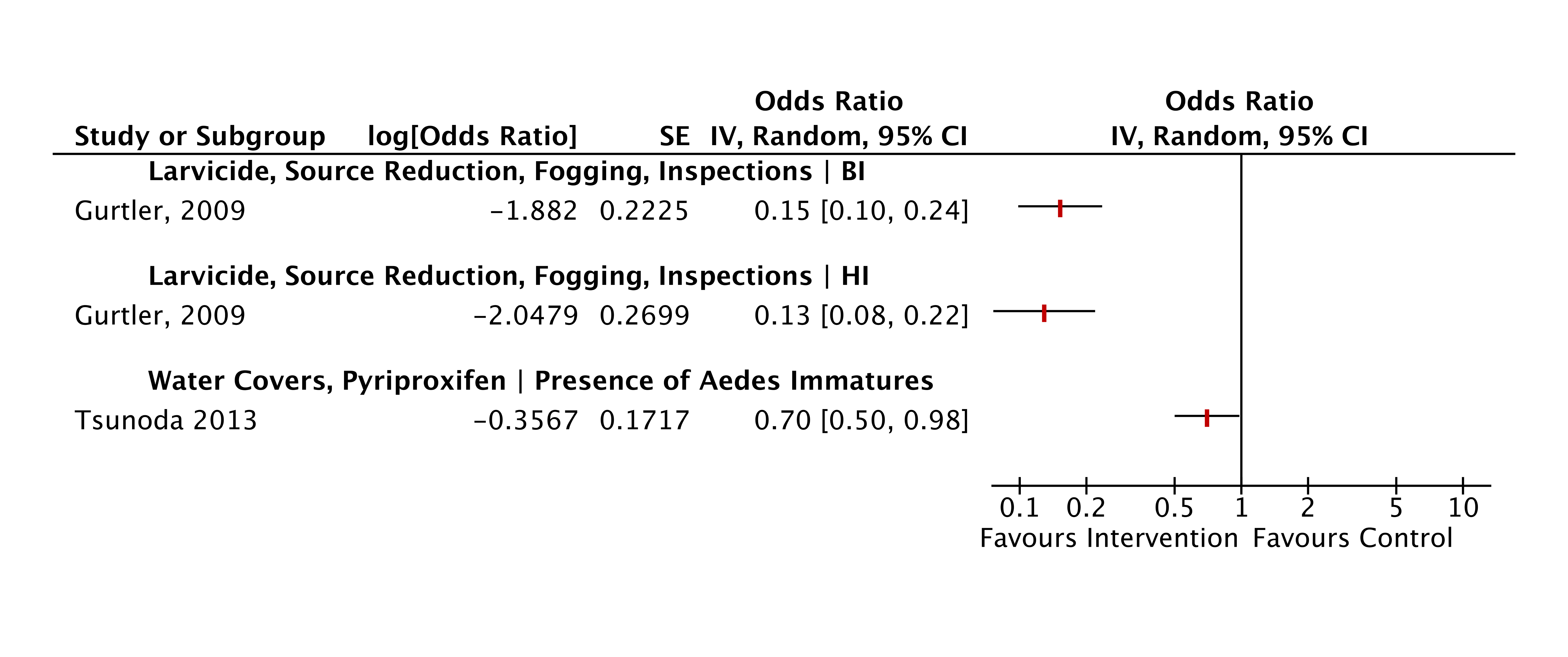

Supplement: S5 Fig — Controlled trial subgroup analysis for larvicide, ULV/ source reduction and Olyset container covers and pyriproxifen vs. control, for the outcomes HI, BI and presence of Aedes sp. immatures stages. (TIF) [file pntd.0004551.s010.tif]

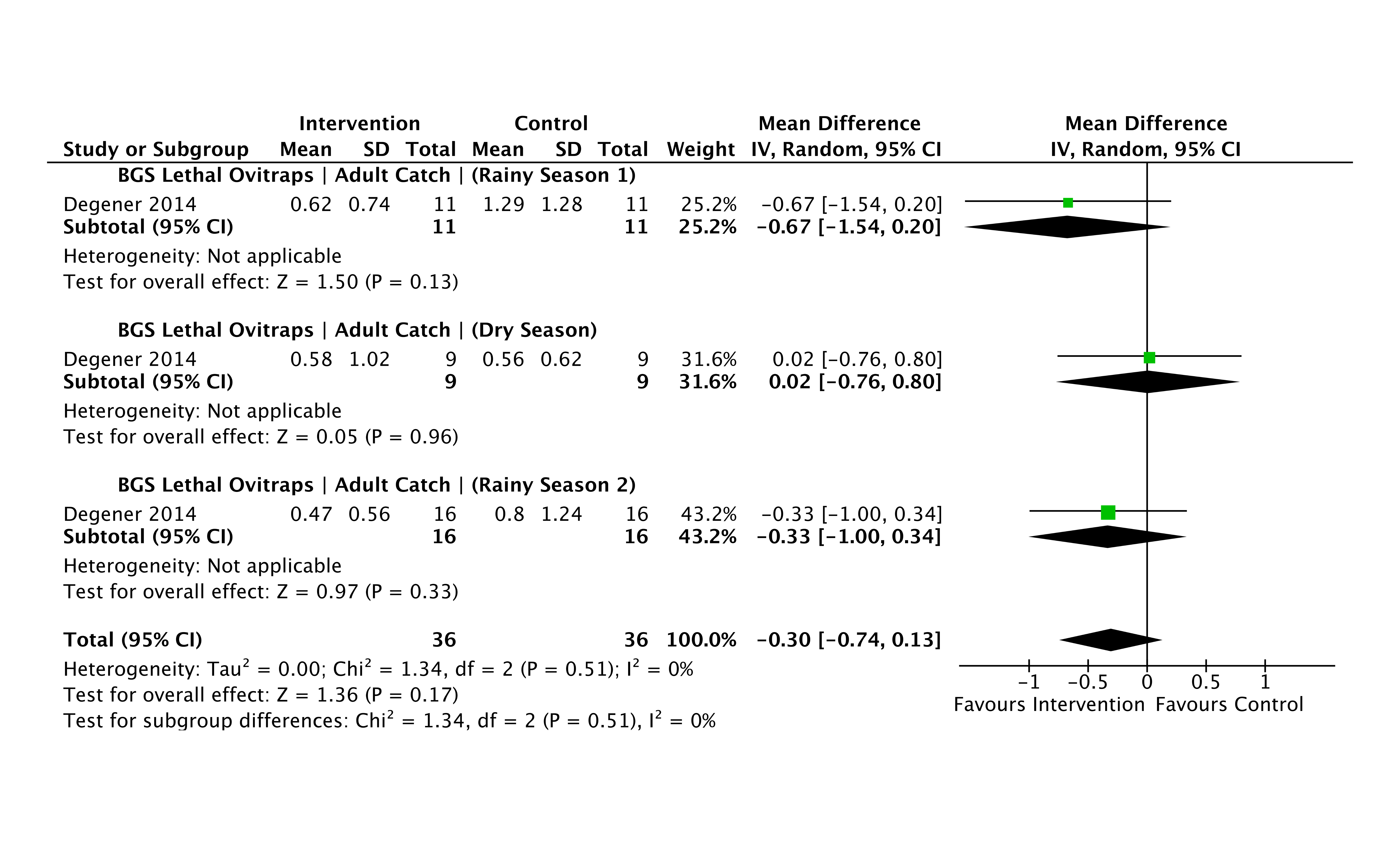

Supplement: S6 Fig — (TIF) [file pntd.0004551.s011.tif]

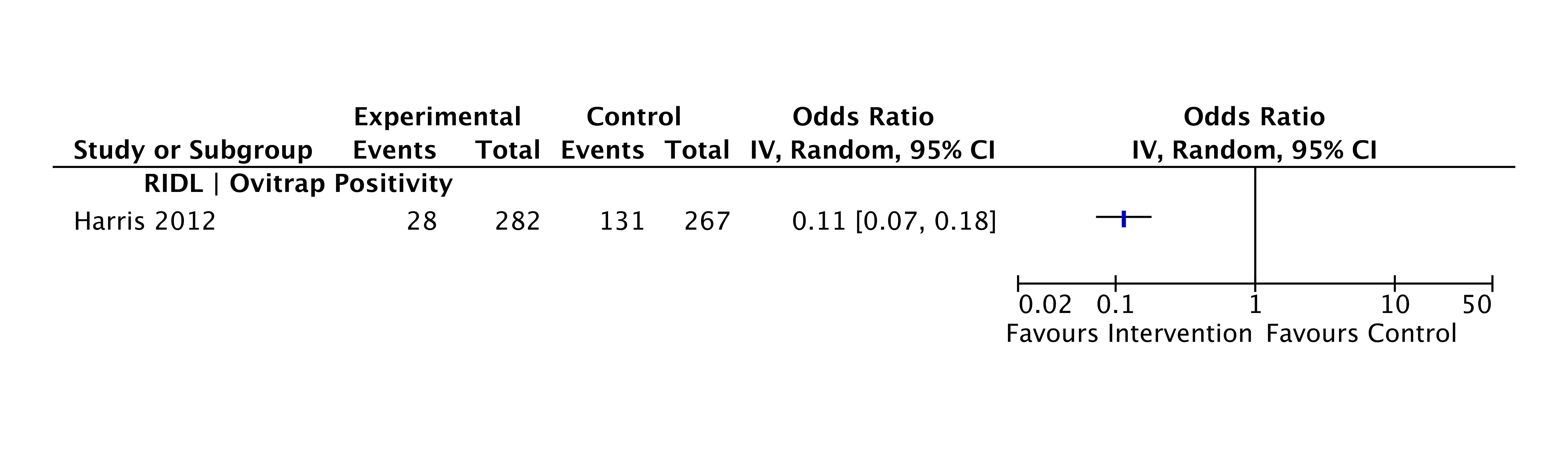

Supplement: S7 Fig — (TIF) [file pntd.0004551.s012.tif]
